# Arthroscopic resection of multiple ossifying tumors in the infrapatellar fat pad

**DOI:** 10.1186/1758-2555-4-43

**Published:** 2012-11-12

**Authors:** Tsutomu Oshigiri, Kota Watanabe, Hidenori Otsubo, Shintaro Takeda, Tomoyuki Suzuki, Takuma Kobayashi, Toshihiko Yamashita

**Affiliations:** 1 The Department of Orthopaedic Surgery, Faculty of Medicine, Sapporo Medical University, 291 nishi-16 minami-1 Chuouku, 060-8543 Sapporo, Chuouku, Japan

**Keywords:** Arthroscopic surgery, Multiple ossifying tumor, Synovial osteochondroma, Infrapatellar fat pad

## Abstract

A 49 year-old male visited a nearby clinic five years back with a complaint of pain in the right knee during exercise. Plain radiographs revealed absence of any anomalies. He began to feel a lumpy mass in his right knee two years back. The pain worsened, on imaging, an anomaly was identified in the infrapatellar fat pad of his right knee, and he was subsequently referred to our department where he was hospitalized. On examination, a mass extending on either side of the patellar tendon was identified along with rigid tenderness in that area. The knee’s range of motion was 0degrees-130degrees, and knee flexion was accompanied by pain. The results of blood tests were normal. A plain radiograph of the knee revealed multiple ossifying tumors at a site consistent with the infrapatellar fat pad. T1-weighted MRI exhibited low-signal intensity, while T2-weighted MRI exhibited a mosaic-shaped tumor. We performed arthroscopic surgery to excise the tumor. The patient resumed work shortly after surgery and did not experience any pain during the two year postoperative observation period. The joint’s range of motion improved to the extent that it was comparable with that of the left knee. No recurrence was observed on radiographic examination. In past studies, resection of similar tumors has been performed with an arthrotomy; however, we performed arthroscopic resection on our patient, who demonstrated a quick improvement in symptoms and range of motion after surgery. We believe that arthroscopic surgery is a feasible option to consider while treating such cases.

## Background

The infrapatellar fat pad is an intracapsular extrasynovial structure. Conditions presenting as ossification of the infrapatellar fat pad are relatively rare and documented in few reports. Helpert et al. [1] have described the MRI characteristics of tumors and tumor-like lesions involving the infrapatellar fat pad; however, preoperative differential diagnosis of such conditions is not necessarily easy. In this paper we report the case of a 49-year-old patient with synovial osteochondroma of the infrapatellar fat pad.

## Case presentation

A 49-year-old man visited a nearby clinic five years back on experiencing pain in his right knee while exercising. Radiographic examination revealed absence of any anomalies. His pain gradually worsened, and two years back, he felt a lumpy mass in his right knee for which he did not seek immediate treatment. The pain continued to worsen, and he eventually visited a nearby clinic, where radiographs revealed an anomaly on the infrapatellar fat pad of his right knee. He was subsequently referred to our department for treatment.

[Family/past medical history]: Not significant.

[Observations on admission]: On examination, a mass accompanied by rigid tenderness and stiffness was identified on either side of the patellar tendon; however, there was no redness or warmth in that area. The knee’s range of motion was slightly restricted (0°–130°), and knee flexion was accompanied by pain.

[Blood/biochemical observations]: Not significant.

**Figure 1:**
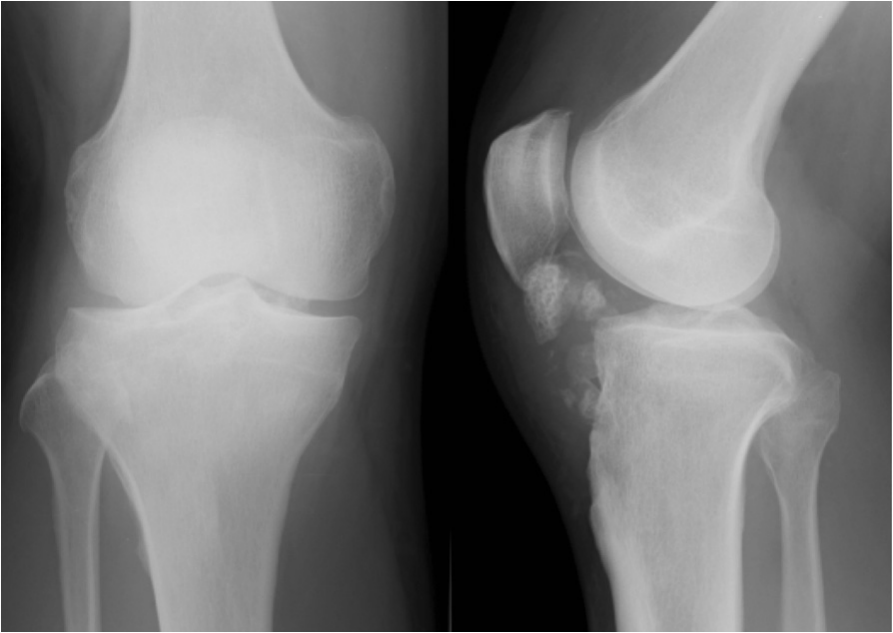
Preoperative radiographs of the left knee showed multiple ossifying tumors at a site consistent with the infrapatellar fat pad.

**Figure 2:**
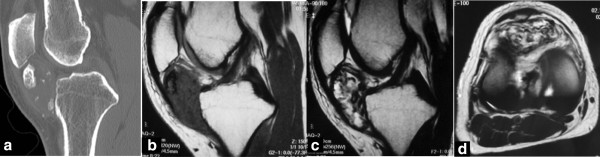
**CT and MRI revealed the multiple tumors as ossification in the infrapatellar fat pad. a** CT MPR image **b** Sagittal T1-weighted MRI **c** Sagittal T2-weighted MRI **d** Axial T2-weighted MRI.

[Imaging observations]: Plain radiographs revealed multiple ossifying tumors at a site consistent with the infrapatellar fat pad (Figure 1). CT scans revealed multiple nodules presenting mineralization varying from some flecks of calcification to ossifying masses in the infrapatellar fat pad (Figure 2a). The lesion exhibited low-signal intensity on T1-weighted MRI, whereas a mosaic-shaped tumor was observed on T2-weighted MRI (Figure 2b-d).

[Surgical observations]: The anterolateral and anteromedial portal were used for arthroscopic surgery. Multiple white tumors of ossifying lesions were observed on the infrapatellar fat pad. We extracted these tumors under arthroscopic guidance using a punch and shaver , and the extraction of major ossifying lesions was carried out piece by piece.

**Figure 3:**
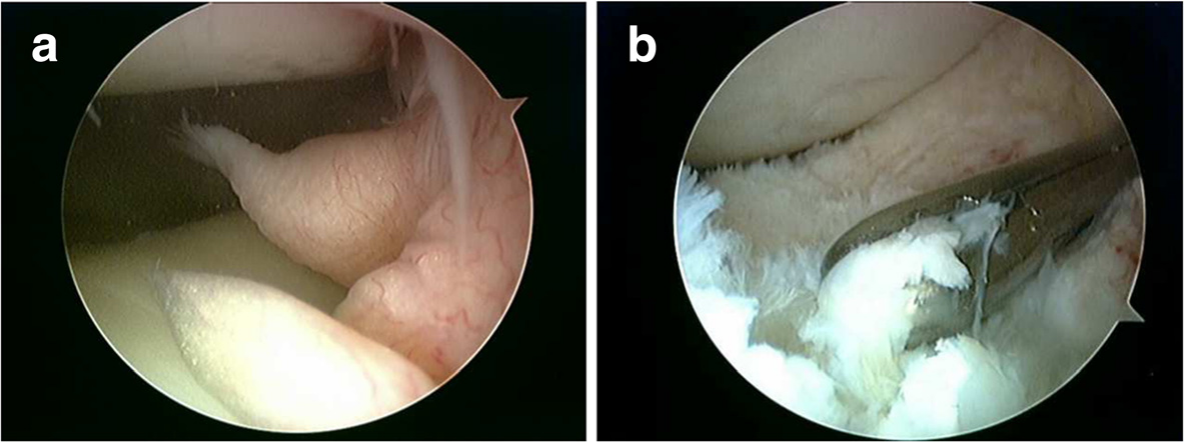
**Findings during arthroscopic surgery.a** Multiple nodules were observed in the infrapatellar fat pad. **b** The fat pad including the tumors were resected arthroscopically with a punch and shaver.

There was no requirement for a large arthrotomy (Figure 3). The surrounding soft tissues were excised along with the tumors, thus exposing the patellar tendon.

**Figure 4:**
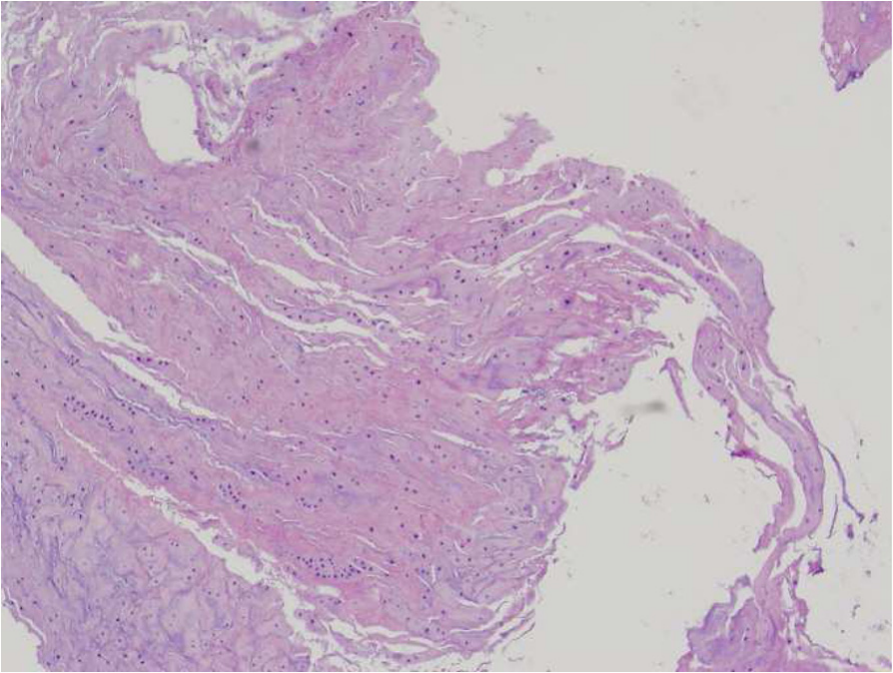
Histological examination confirmed the diagnoses of osteochondroma (hematoxylin-eosin stain).

[Pathological findings]: Synovial membrane tissue was identified in the excised specimen. In addition, fatty bone marrow, which marks a transition to endochondral ossification, was observed in addition to an ossification nest. A pathological diagnosis of synovial osteochondromatosis was made (Figure 4).

**Figure 5:**
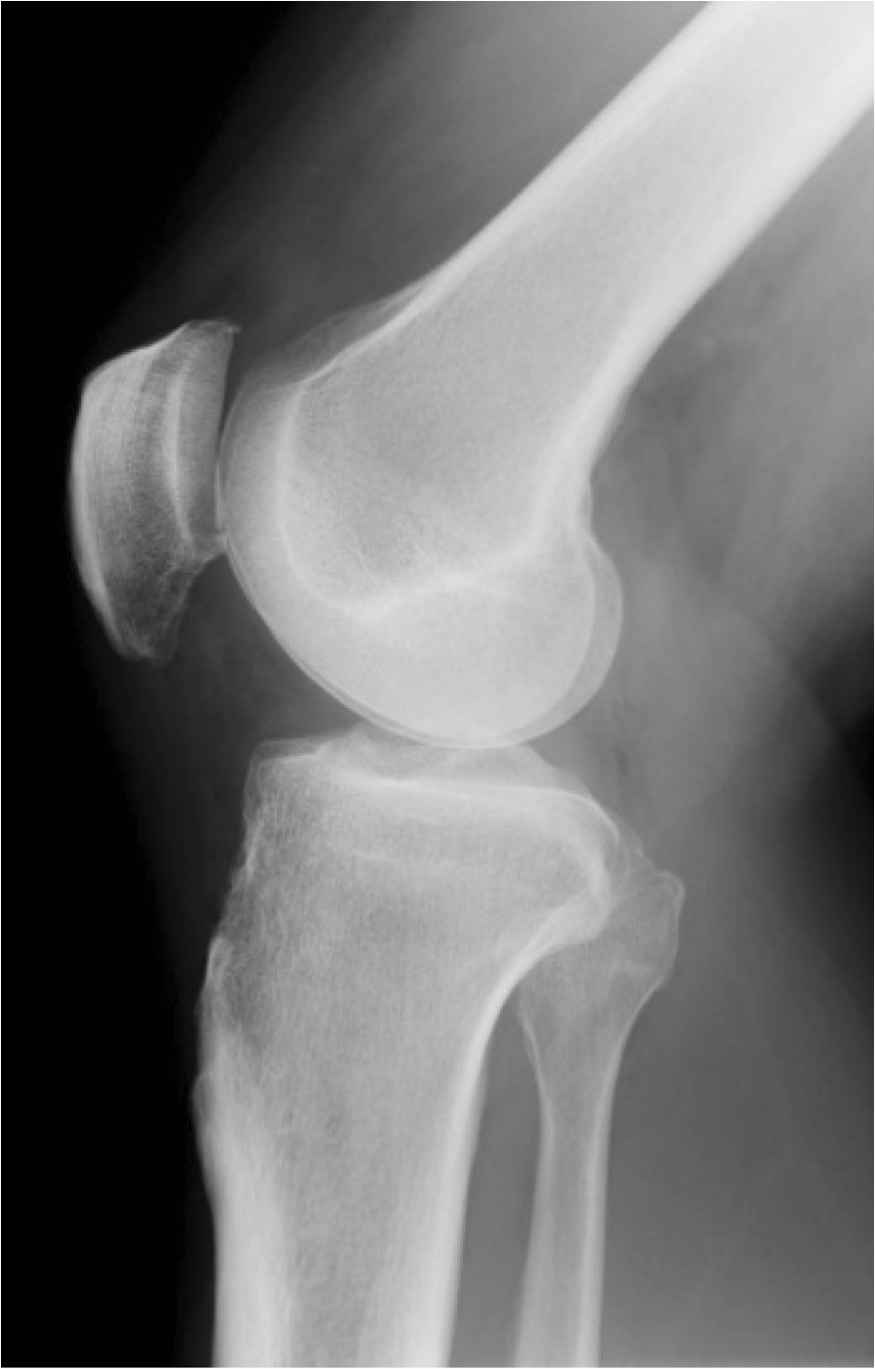
**Radiographs 2 years after the operation.** No signs of recurrence were observed.

[Postoperative course]: The patient was permitted to walk with crutches from the immediate postoperative day, provided the load on the knee was tolerable. Nevertheless, his knee was able to bear a full load within a short time after surgery. The range of motion in the operated knee quickly improved to preoperative levels and reached 0°–145° one year after surgery. The patient did not experience any pain during the two-year postoperative observation period. The right knee joint’s range of motion improved to the extent that it was comparable with that of the left knee joint. No signs of recurrence were observed on follow-up radiographic examination (Figure 5).

## Discussion

Diseases presenting as ossification of the infrapatellar fat pad are relatively rare. Paraarticular osteochondroma, angioleiomyoma, myositis ossificans, crystal deposition and, in malignant conditions, extraskeletal osteosarcoma and chondrosarcoma, are considered as differential diagnoses in addition to synovial osteochondroma [2,3]. If malignant diseases are suspected, it is necessary to perform further examinations such as contrast MR imaging and thallium scintigraphy. The imaging findings of these benign conditions are similar, thus preoperative differential diagnosis is often difficult. Given the origin of occurrence from tissue, extraarticular synovial osteochondromatosis has been considered to be distinct from the juxtacortical, paraarticular, or intracapsular osteochondroma [4]. The differentiation is based on the identification of a definite synovial origin for the former. Primary symptoms in all these conditions include the presence of a lumpy mass accompanied by discomfort and pain. In addition, the mass grows at a slow rate. Reith et al. [2] combined the results of past reports with his own to prepare a report on the characteristics of paraarticular osteochondroma. He pointed out that precise judgment, which is based on both imaging and histolgic findings, is required to differentiate paraarticular osteochondroma from other osteochondral lesions and to obtain a definitive diagnosis. Maeno et al. [3] reported the excision of an angioleiomyoma, which was accompanied by calcification on the lower edge of the patella as observed on a plain radiograph. Angioleiomyoma reportedly has a predilection for the lower limbs of middle-aged patients, and is often accompanied by a characteristic pain. The pain is often paroxysmal and is initiated even by lightly touching the tumor, by exposure of the tumor to wind or cold, and by other imperceptible stimuli. Calcification of an angioleiomyoma is reportedly rare (2%–10%); however, it should be considered during the differential diagnoses of related conditions [5]. Similar to crystal deposition and myositis ossificans, it is often accompanied by pain; furthermore, a history of conditions like gout, trauma, etc., would serve as a useful reference for diagnosis.

The lesion we experienced was diagnosed as synovial chondromatosis of the infrapatellar fat pad because it was identified as synovial in origin. Helpert et al. [1] reported twelve cases of primary synovial chondromatosis of the knee. Six (50%) had irregularity with invasion/replacement of the infrapatellar fat pad. In none of the cases was the disease confined to the infrapatellar fat pad. All showed synovial proliferation with soft tissue masses in the suprapatellar pouch and/or popliteal fossa. It is considered that synovial osteochondroma rarely arises in the infrapatellar fat pad. Benign lesions have been treated by excision or marginal resection via arthrotomy [6-12]. In this case, we were able to identify the extent of the tumor on MRI and subsequently resect it under arthroscopic guidance. To our knowledge, there are no reports of arthroscopic resection surgery for synovial osteochondroma of the infrapatellar fat pad. In our study, surrounding soft tissues were excised along with the tumors, and exposure of the patellar tendon was confirmed by arthroscopy. Range of motion normalized shortly after surgery and was comparable with that of the left knee within one year. The patient did not experience any pain during the two-year postoperative observation period; moreover, no signs of recurrence were seen on plain follow-up radiographs. Our findings indicate that in future, arthroscopic surgery should be considered as a feasible treatment option for conditions like those reported in this study.

## Consent

Written informed consent was obtained from the patient and the patient’s parents for publication of this Case report and any accompanying images. A copy of the written consent is available for review by the Editor-in –Chief of this journal.

## Competing interests

The authors declare that they have no conflict of interest.

## Authors’ contributions

All authors co-wrote the paper and discussed the results for the manuscript preparation. All authors have read and approved the final manuscript.
